# Supplementary material for: Cryo-EM structures of the MnmE–MnmG complex reveal large conformational changes and provide new insights into the mechanism of tRNA modification
Source: Nucleic Acids Res. 2025 Aug 30;53(16):gkaf824. doi: 10.1093/nar/gkaf824 (PMC12397908; doi:10.1093/nar/gkaf824)
Supplement: gkaf824_Supplemental_Files [file gkaf824_supplemental_files.zip › Maes_etal_MnmG_SI_revision.pdf]

Supplementary Information

Cryo-EM structures of the MnmE-MnmG complex reveal large conformational changes and provide new insights into the mechanism of tRNA modification

Laila Maes[1,2], Israel Mares-Mejía[1,2], Ella Martin[1,2], David Bickel[1,3,*], Siemen Claeys[1,2], Wim Vranken[1,3], Marcus Fislage[1,2], Christian Galicia[1,2,$], Wim Versées[1,2,$]

[1] *Structural Biology Brussels, Vrije Universiteit Brussel, Pleinlaan 2, 1050 Brussels, Belgium.*
[2] *VIB-VUB Center for Structural Biology, VIB, Pleinlaan 2, 1050 Brussels, Belgium.*
[3] *Interuniversity Institute of Bioinformatics in Brussels, ULB-VUB, Brussels, Belgium.*

[$] *To whom correspondence should be addressed. Tel: +32-2-6291849; Email: wim.versees@vub.be. Correspondence may also be addressed to Christian Galicia. Email: cgalicia@vub.be.*

[*] *Current address: SANTERO Therapeutics, 1435 Mont St Guibert, Belgium*

SUPPLEMENTARY FIGURES

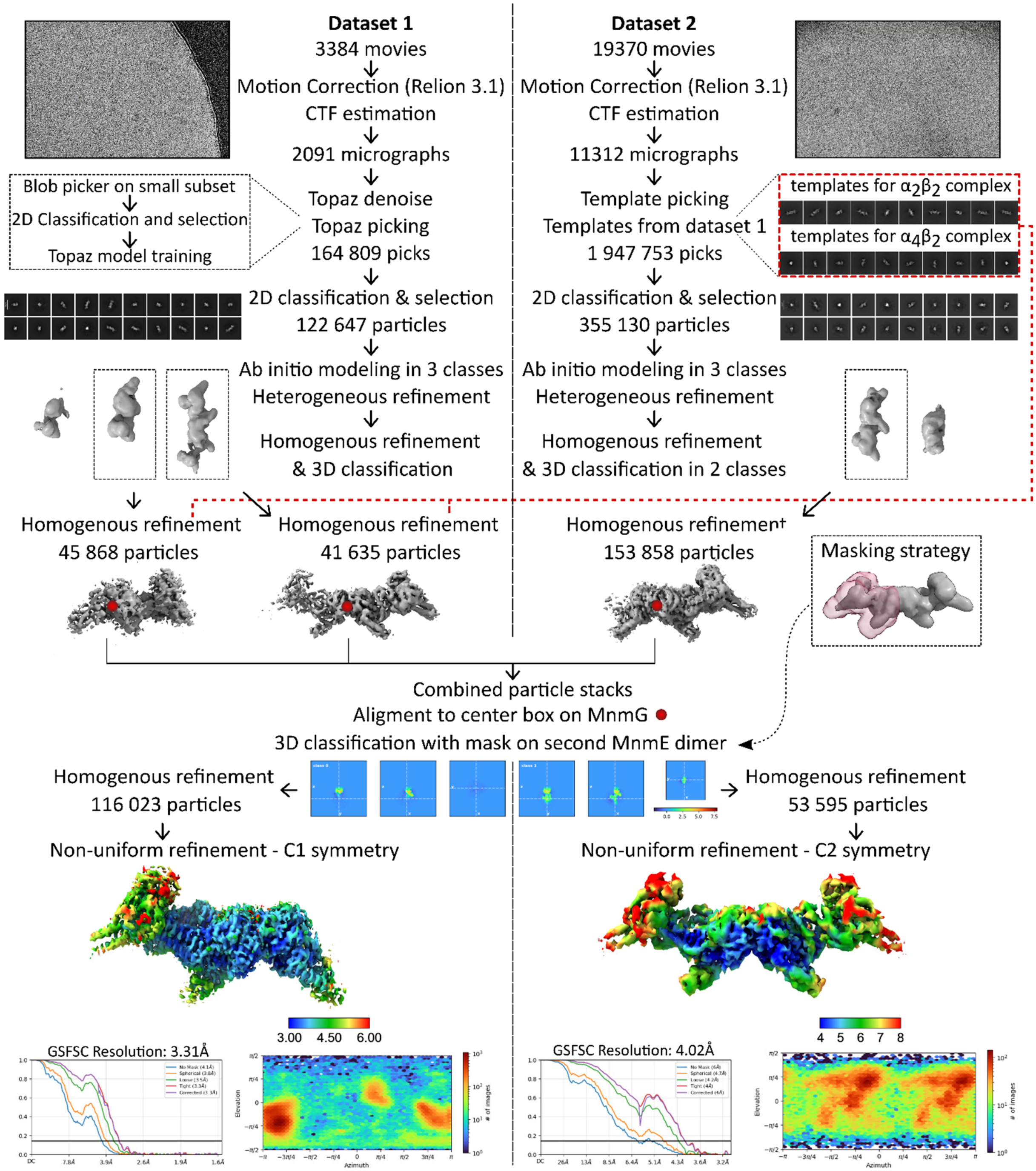

Figure S1. Cryo-electron microscopy (cryo-EM) workflow to obtain the maps corresponding to the $\alpha_2\beta_2$ and $\alpha_4\beta_2$ MnmEG complexes. Flow chart of the cryo-EM data processing for both datasets, including picking strategies, 2D classification, 3D classification with mask and density map reconstructions. Local resolution maps, Fourier shell correlation (FSC) curves, particle distribution plots, and cryo-EM density maps are also shown. All analyses were performed using cryoSPARC v3.3, unless stated otherwise. Details are provided in the 'Materials & Methods' section.

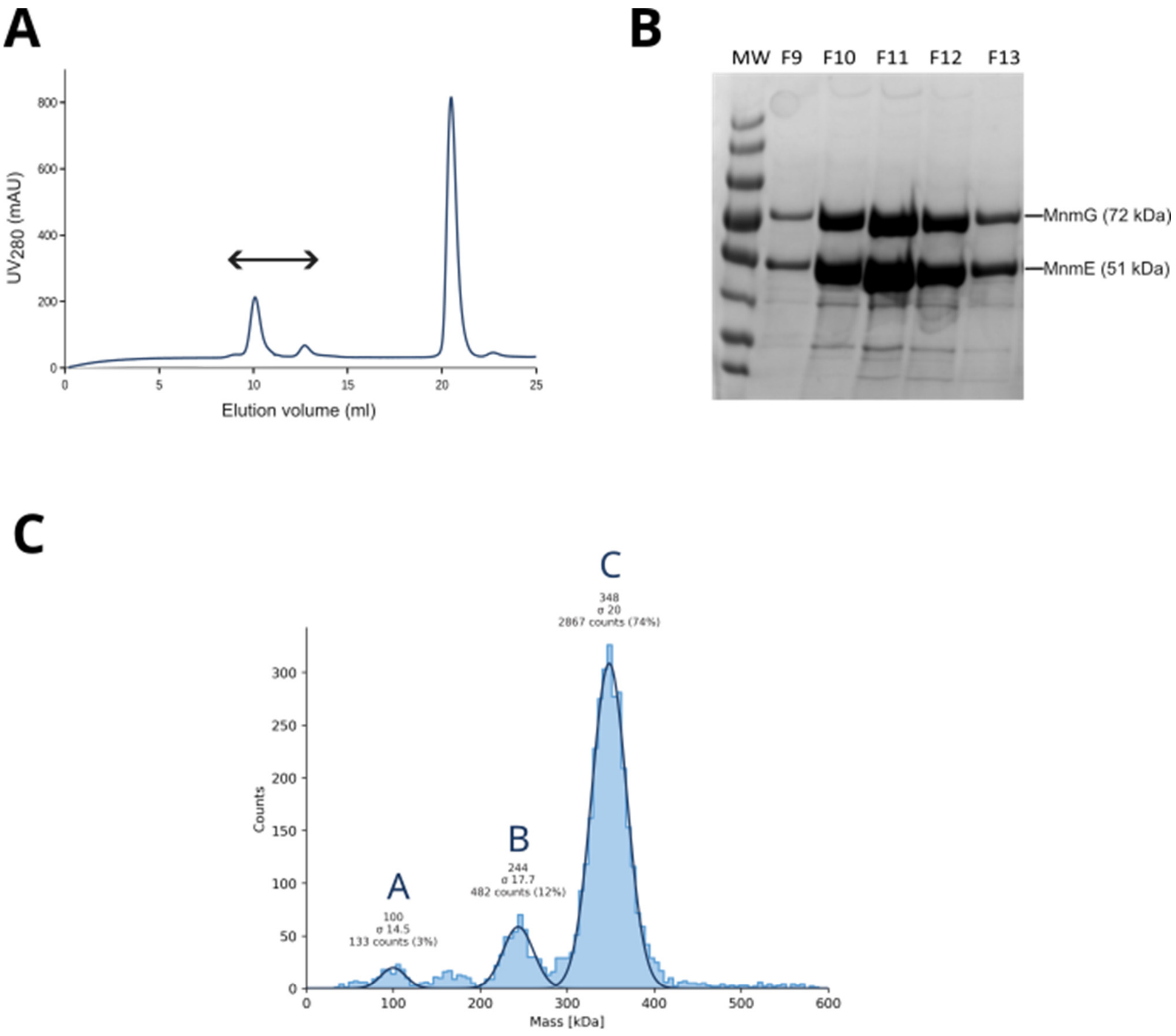

Peak	Molecular weight (kDa)	Molar fraction	Counts	Distribution (σ)
A	100	0.03	133	14.5
B	244	0.12	482	17.7
C	348	0.74	2867	20

Figure S2. Purification and analysis of the MnmEG complex. (A) Purification of the MnmEG complex on size exclusion chromatography (SEC). 100 µM MnmE and 50 µM MnmG were mixed in the presence of 1 mM GppNHp and 1 mM FAD, and the mixture was loaded on a Superdex 200 10/300 column to separate the $\alpha_4\beta_2$ complex. Fractions that were analyzed on SDS-PAGE are indicated with an arrow. **(B)** SDS-PAGE analysis of the peak fractions from SEC corresponding to the $\alpha_4\beta_2$ complex. Fraction 10 and 11 (F10-F11) were pooled for analysis by mass photometry. (**C**) Mass photometry analysis of the pooled fractions 10-11 at a concentration of 100 nM on a Refeyn OneMP instrument. This analysis indicates that more than 70% of the population attains a molecular mass close to the 348 kDa expected for the $\alpha_4\beta_2$ complex.

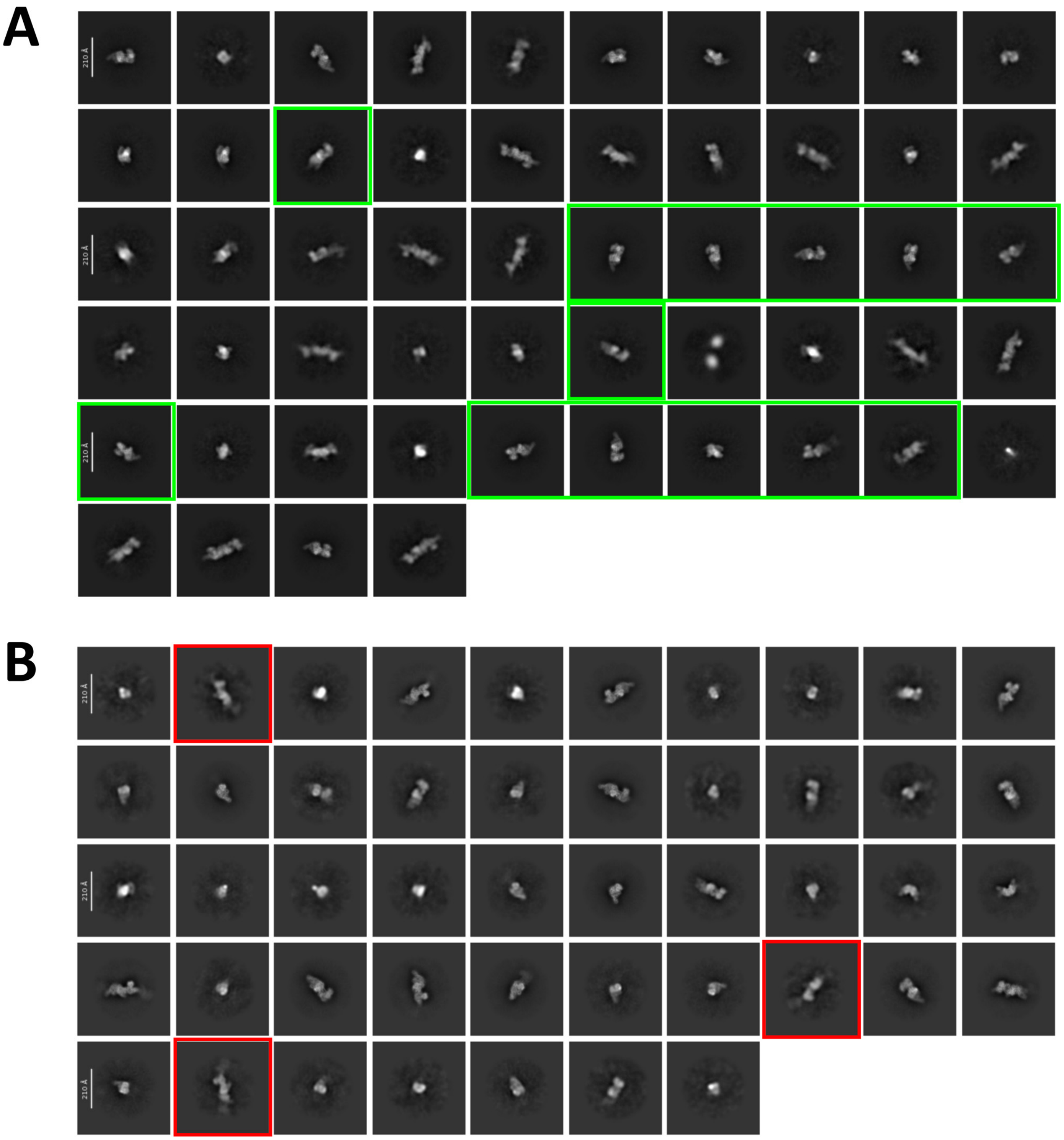

Figure S3. Representative 2D classes of the MnmEG complexes in the two cryo-EM datasets that were collected. **(A)** 2D classes from particles extracted in dataset 1 with a box size of 504 Å. Most 2D classes represent elongated particles corresponding to the $\alpha_4\beta_2$ complex. Nevertheless, in some classes (indicated with a green square) a slightly less elongated particle is observed, indicating that also the smaller $\alpha_2\beta_2$ oligomeric state is present. **(B)** 2D classes from particles extracted in dataset 2 with a box size of 504 Å. Although most classes represent the smaller $\alpha_2\beta_2$ oligomeric state, some classes display a blurred density representing the presence of a second MnmE dimer in the $\alpha_4\beta_2$ complex (indicated by red squares).

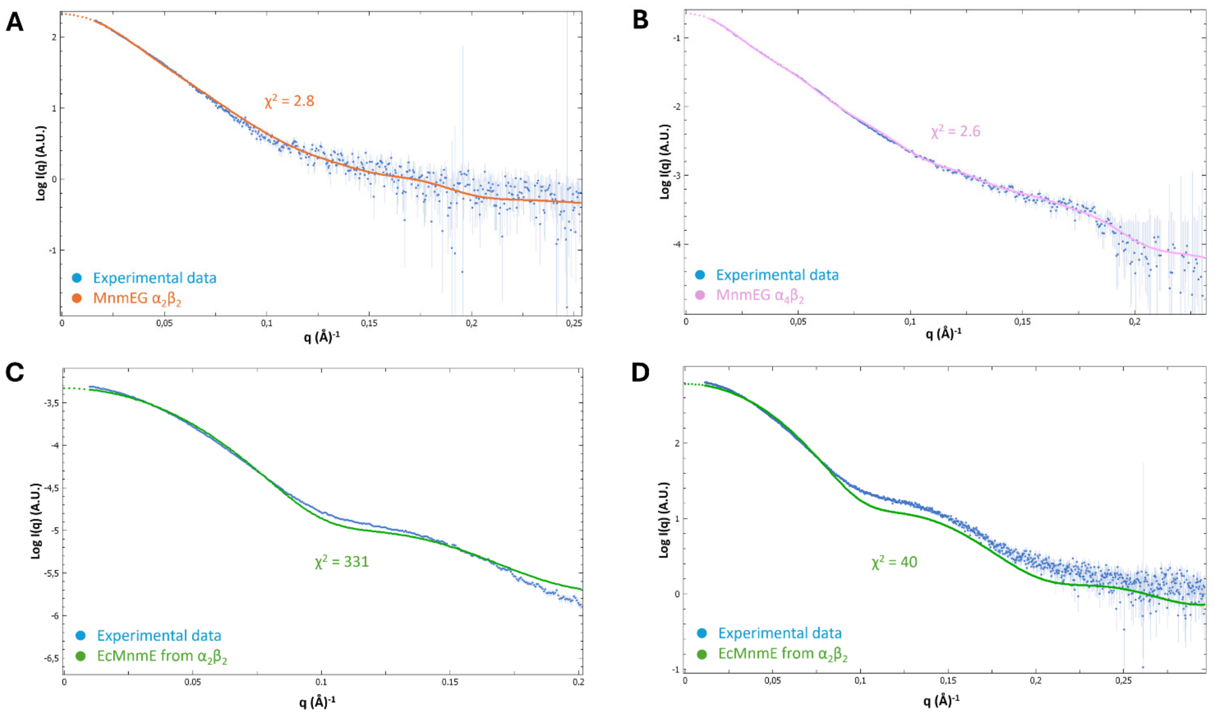

Figure S4. **Comparison of the cryo-EM models of the $\alpha_2\beta_2$ and $\alpha_4\beta_2$ complexes with prior obtained solution SAXS data using Crysol** (see *Fislage et al. Nucleic Acids Res. 42(9):5978-92, 2014)* [1]. **(A)** Superposition of experimentally obtained SAXS data for the $\alpha_2\beta_2$ complex (blue) and the theoretical scatter curve recalculated from the cryo-EM model of the $\alpha_2\beta_2$ complex (red). The experimental scattering curve agrees well with the theoretical scattering curves obtained from the model, with a χ^2 value of 2.8. **(B)** Superposition of experimentally obtained SAXS data for the $\alpha_4\beta_2$ complex (blue) and the theoretical scatter curve recalculated from the cryo-EM model of the $\alpha_4\beta_2$ complex (purple). The experimental scattering curve agrees well with the theoretical scattering curves obtained from the model, with a χ^2 value of 2.6. **(C/D)** Superposition of the theoretical scatter curve calculated from the asymmetric MnmE dimer extracted from the $\alpha_2\beta_2$ complex (green) and the experimentally obtained SAXS data (blue) for MnmE bound to GDP*AlF_4 (**C**) or GppNHp (**D**). This analysis shows significant differences in the overall shape of GppNHp- or GDP*AlF_4-bound MnmE in solution and the conformation of MnmE in complex with MnmG. For all details on the conditions of the experimentally obtained scatter curves see: *Fislage et al. Nucleic Acids Res. 42(9):5978-92, 2014* [1].

A

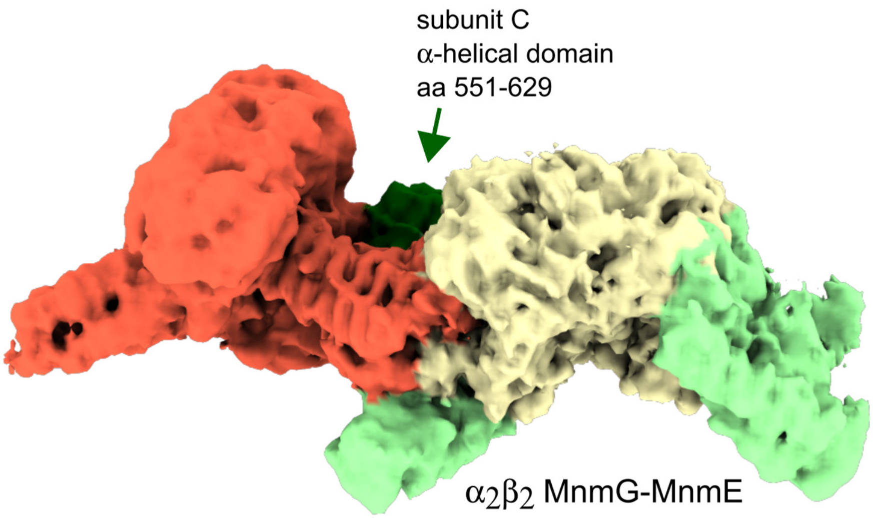

B

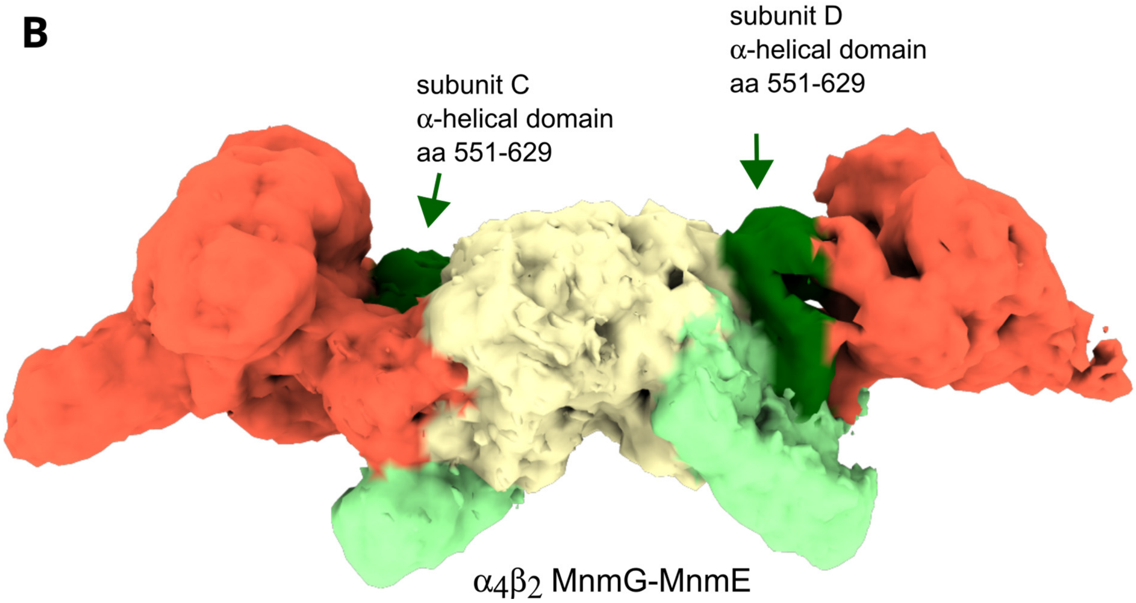

Figure S5. Cryo-EM maps of the MnmEG $\alpha_2\beta_2$ and $\alpha_4\beta_2$ complexes without imposing symmetry. The maps of MnmE are colored red. The map of MnmG is colored yellow, with the N-terminal part of its α-helical domains colored pale green and the C-terminal part of its α-helical domains (551-629) colored dark green. Both maps represent full reconstructions without any imposed symmetry.

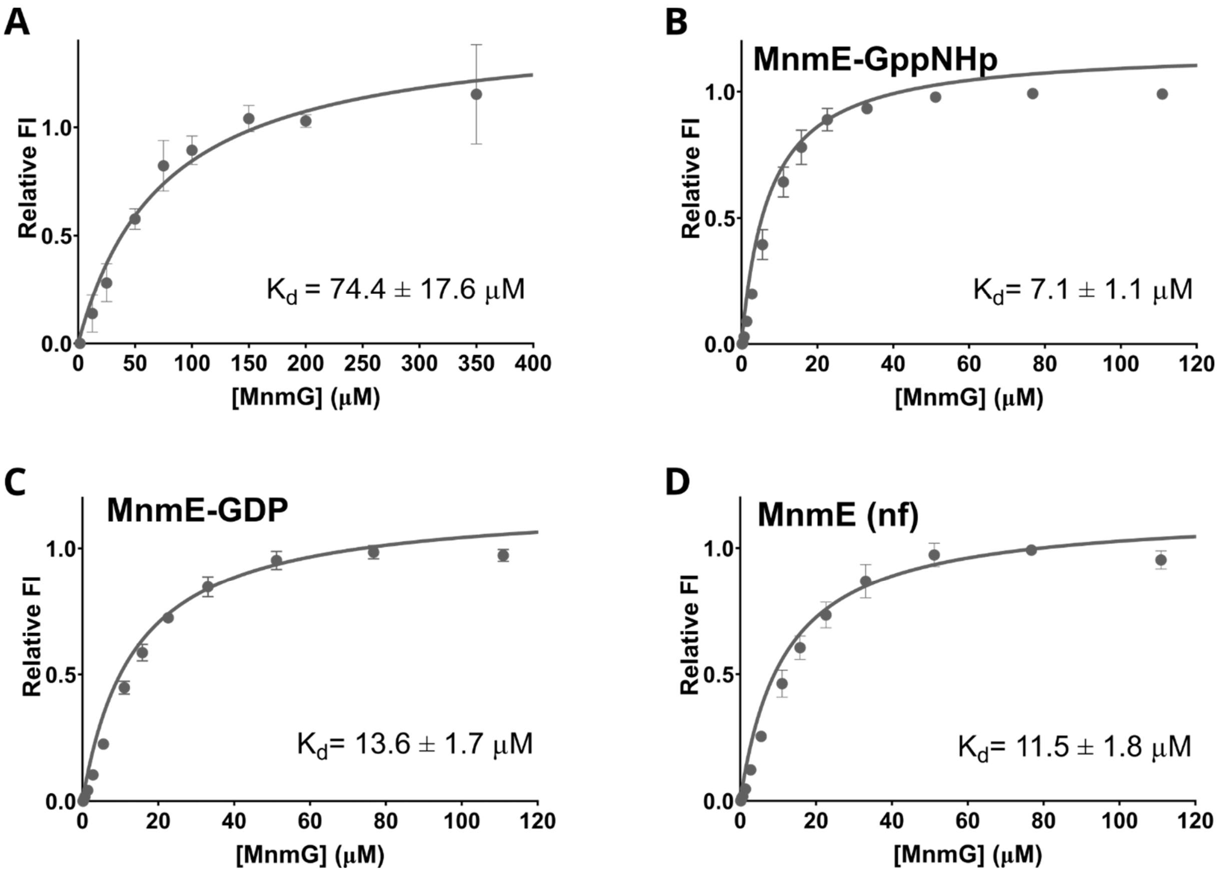

Figure S6. MnmE affects the binding affinity of MnmG for FAD. The binding affinity (K_D) of MnmG for FAD was determined by measuring the intrinsic fluorescence of FAD at different concentrations of MnmG, either in absence of MnmE (**A**), or in presence of an excess of MnmE bound to GppNHp (**B**), GDP (**C**) or in a nucleotide-free (nf) state (**D**). All experiments were performed in triplicate. To obtain the corresponding K_D values (± standard error), the data were fitted using the quadratic binding equation in GraphPad Prism 7.

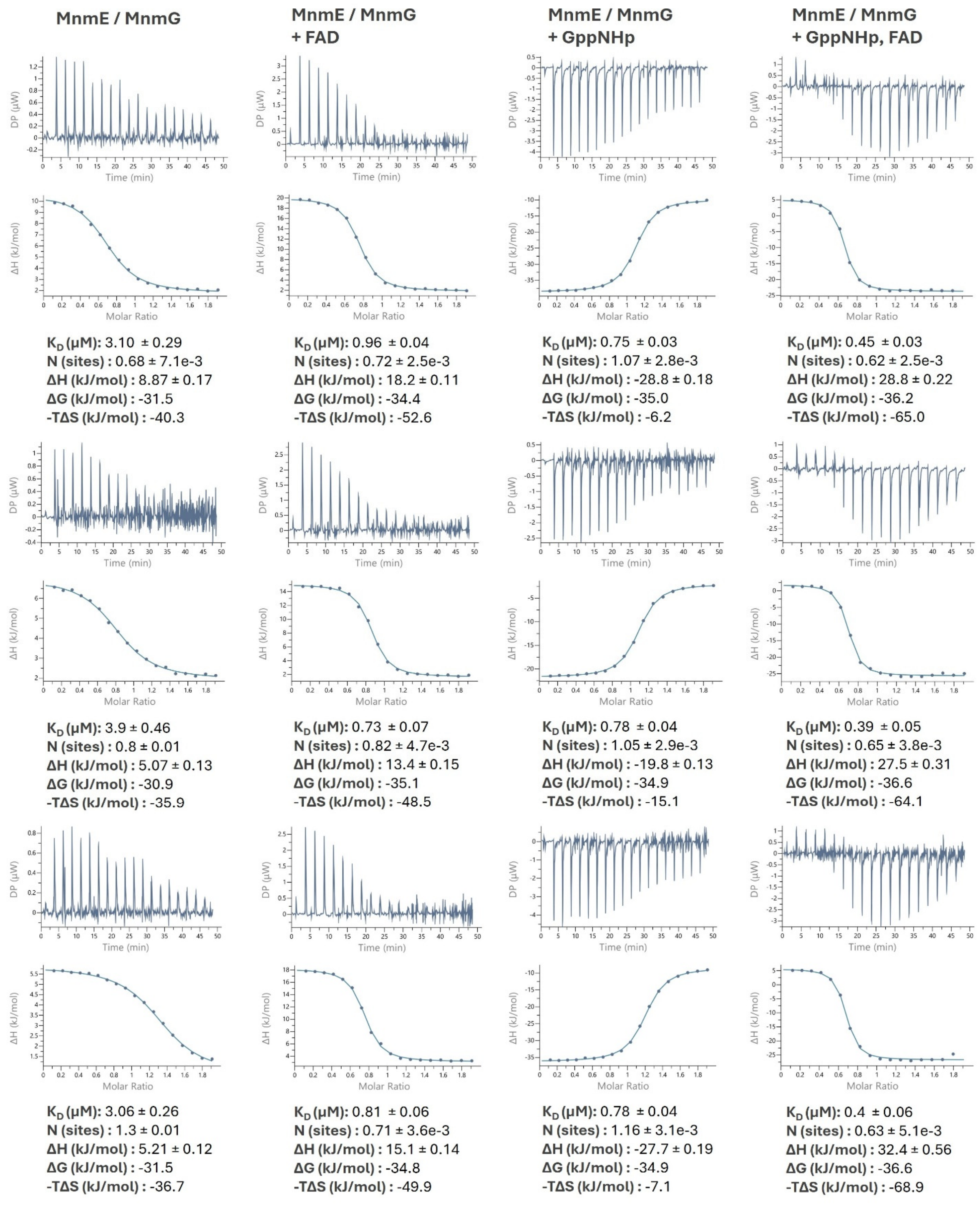

Figure S7. ITC measurements to assess the binding between MnmG (in the cell at 70 μM) and MnmE (in the syringe at 700 μM). The binding is measured in the absence and presence of either FAD (1 mM), GppNHp (1 mM) or both. Each of these conditions was measured in triplicate, with the corresponding individual curves shown underneath each other. The resulting binding isotherms were fitted on a "one set of sites" model to determine affinities (K_D), stoichiometries (n) and the corresponding thermodynamic parameters (ΔG, ΔH, $-T\Delta S$).

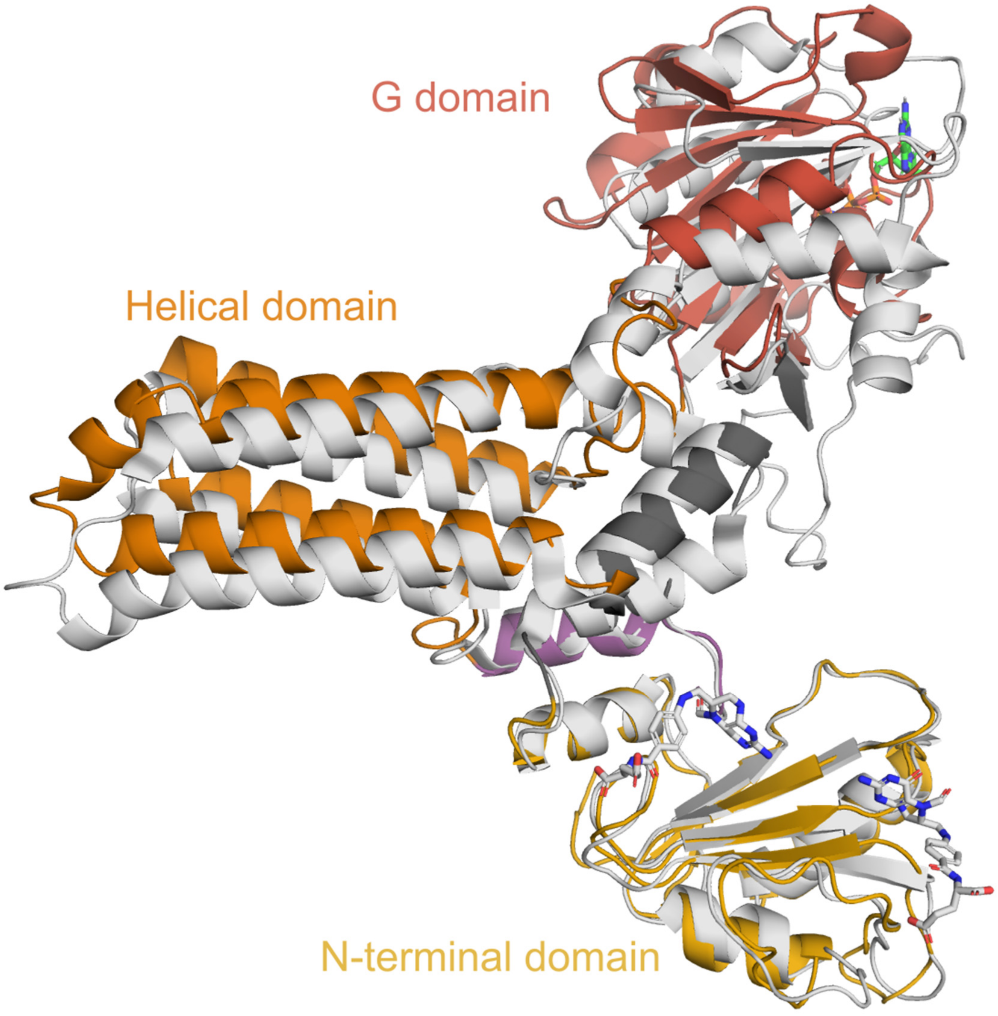

Figure S8. Superposition of the A subunit of MnmE extracted from the $\alpha_2\beta_2$ complex on a subunit of the crystal structure of MnmE from *Thermatoga maritima* (TmMnmE, PDB 1XZQ). The domains of the A subunit of MnmE from the $\alpha_2\beta_2$ complex are indicated with the N-terminal domain, helical domain and G domain in yellow, orange and red, respectively, while the hinge helix and swivel helix are shown in dark grey and purple, respectively. The subunit of TmMnmE is shown in grey.

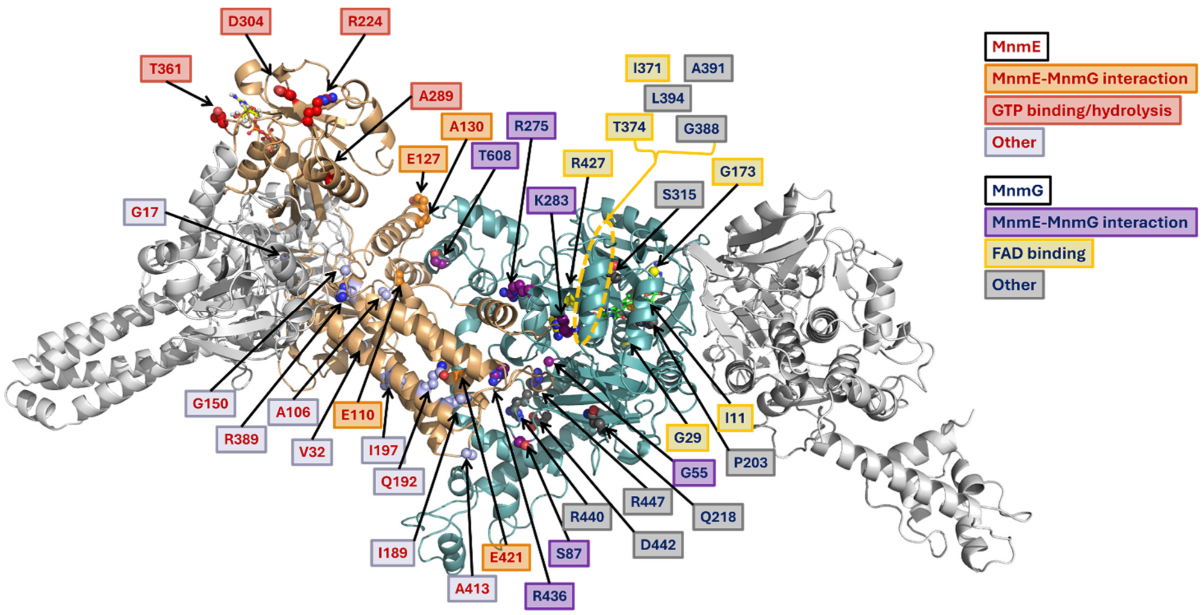

Figure S9. MnmE and MnmG residues corresponding to known disease mutations in their human orthologues GTPBP3 and MTO1, respectively, mapped on the cryo-EM structure of the MnmE-MnmG $\alpha_2\beta_2$ complex. MnmE and MnmG are shown in cartoon representation, with the A and B chains of MnmE colored grey and light orange, respectively, and the C and D chains of MnmG colored cyan and grey respectively. The residues corresponding to known disease mutations are mapped on the interacting MnmE B chain and MnmG C chain, shown in sphere representation and colored according to their assumed functional role in the tRNA modification reaction, as previously reported and/or as inferred from the current structure. The color code used is indicated next to the figure. An overview of all these disease mutations and the associated pathologies, together with the citations to the corresponding references, is given in **Table S2**.

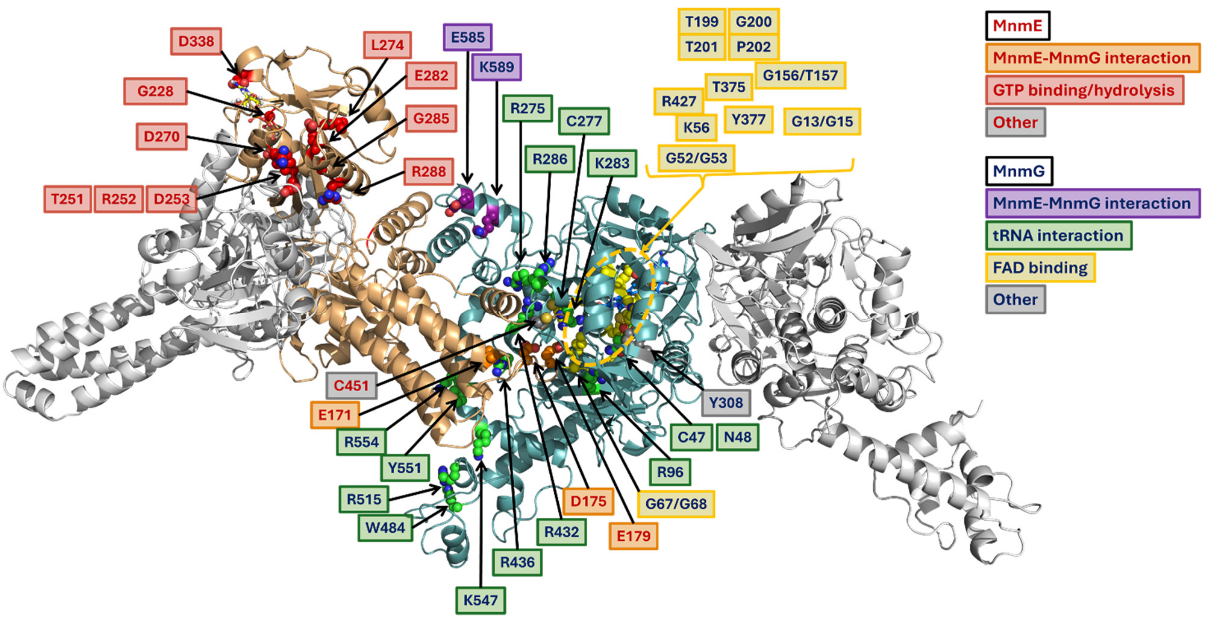

Figure S10. MnmE and MnmG residues with previously reported roles in tRNA binding and / or tRNA modification mapped on the cryo-EM structure of the MnmE-MnmG $\alpha_2\beta_2$ complex. MnmE and MnmG are shown in cartoon representation, with the A and B chains of MnmE colored grey and light orange, respectively, and the C and D chains of MnmG colored cyan and grey respectively. The residues with reported roles in tRNA binding or modification are mapped on the interacting MnmE B chain and MnmG C chain, shown in sphere representation and colored according to their functional role in tRNA modification, as previously reported and/or as inferred from the current structure. The color code used is indicated next to the figure. An overview of all these residues with reported roles in tRNA binding / modification, with the citations to the corresponding references, is given in **Table S3**.

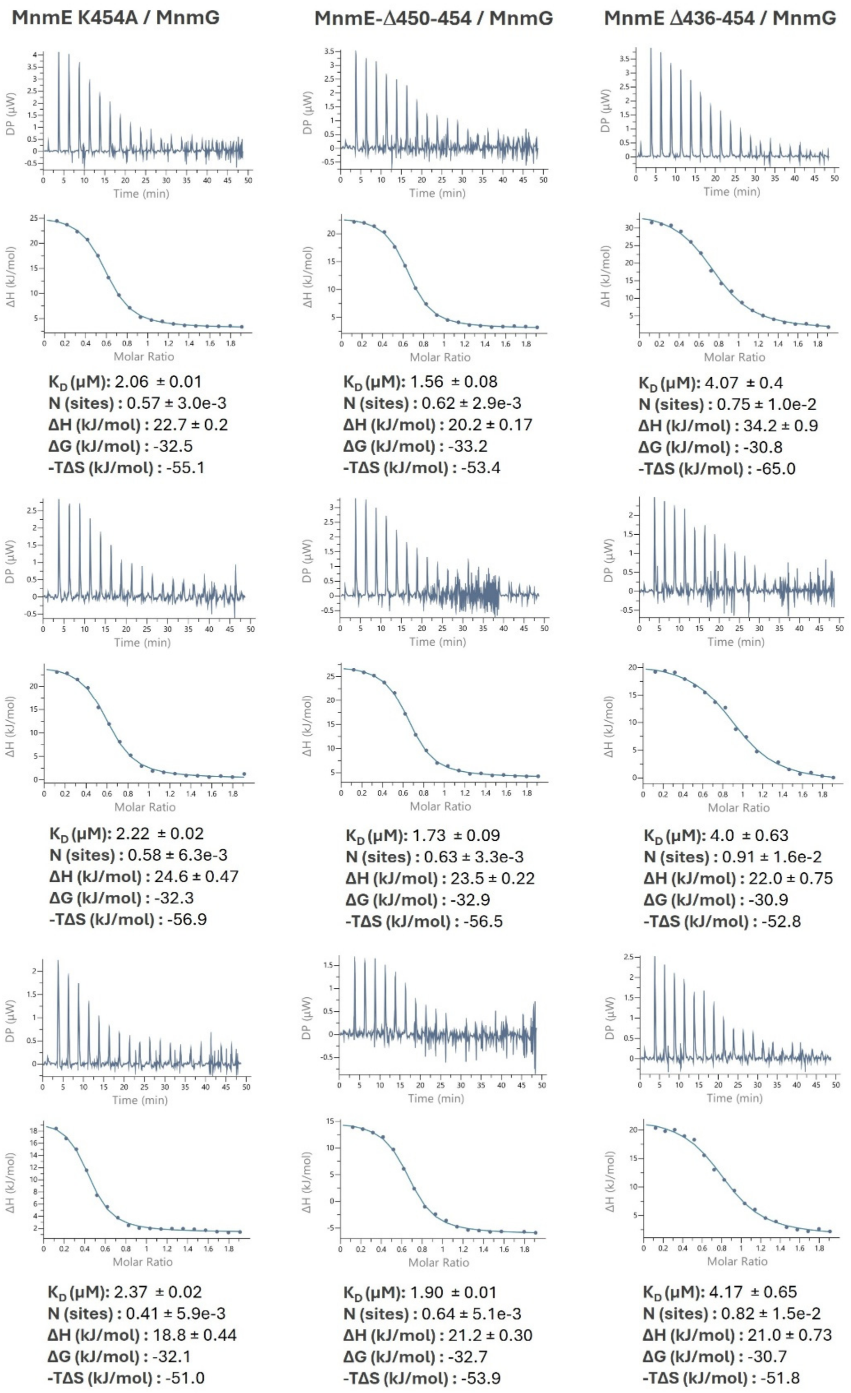

Figure S11. ITC measurements to assess the binding between MnmG (in the cell at 70 µM) and the K454A, ∆450-454 and ∆436-454 MnmE mutants (in the syringe at 700 µM), in the presence of both FAD (1 mM) and GppNHp (1 mM). Each of these conditions was measured in triplicate, with the corresponding individual curves shown underneath each other. The resulting binding isotherms were fitted on a "one set of sites" model to determine affinities (K_D), stoichiometries (n) and the corresponding thermodynamic parameters (∆G, ∆H, -T∆S).

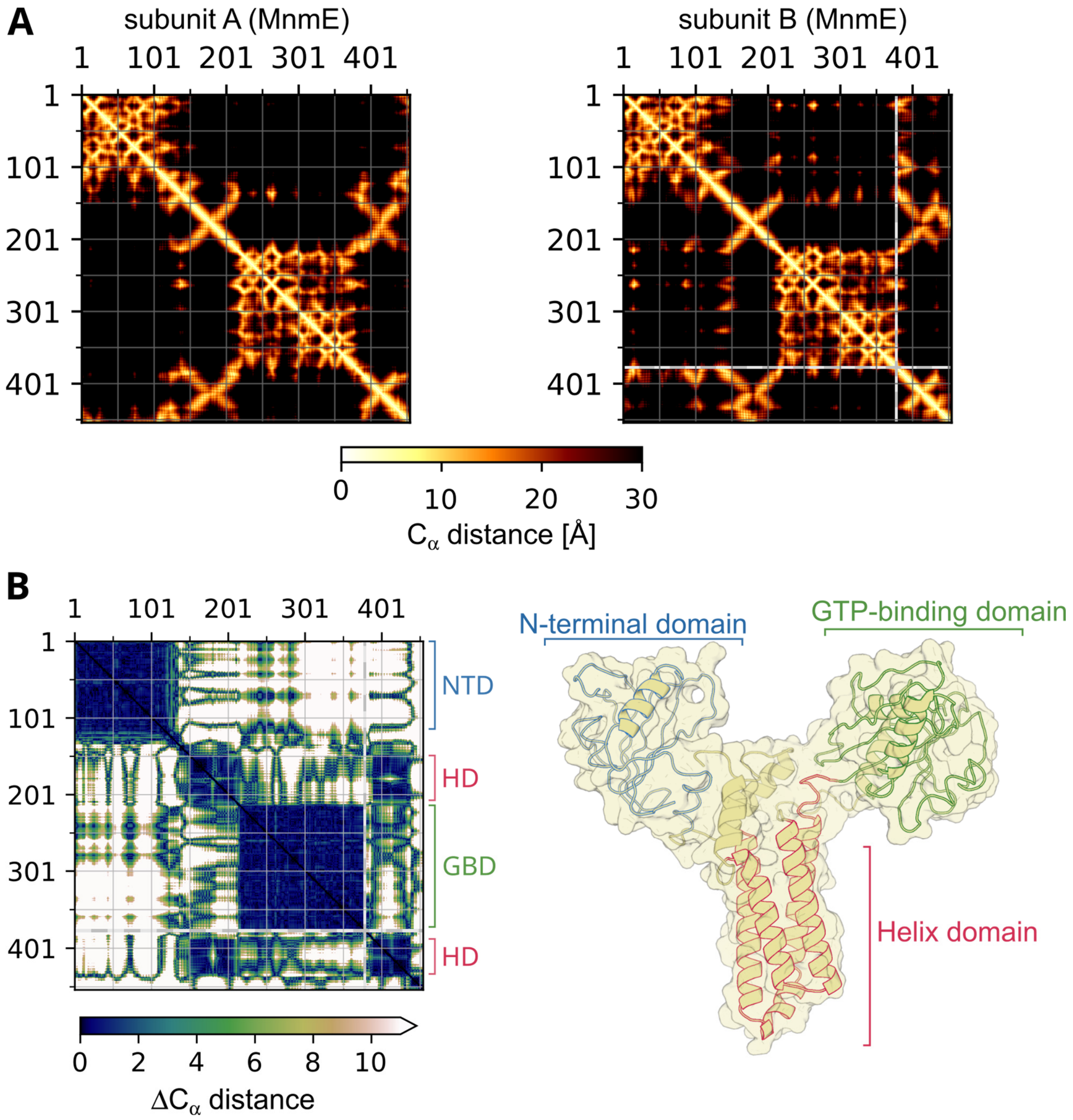

Figure S12: **Analysis and comparison of the Cα distance matrices of the MnmE subunits A and B in the $\alpha_2\beta_2$ model.** (**A**) Cα distance matrix of MnmE subunit A (*left*) and subunit B (*right*). Unresolved residues are shown in a grey checkerboard pattern. (**B**) Difference in the Cα-Cα distance matrices between the subunit A and subunit B conformations of MnmE (*left*) and mapping of the domains that act as rigid bodies on the subunit structure of MnmE. It can be observed that the N-terminal domain (NTD), G domain (GBD) and the four-helix bundle of the helical domain (HD) move relative to each other but retain their internal conformation.

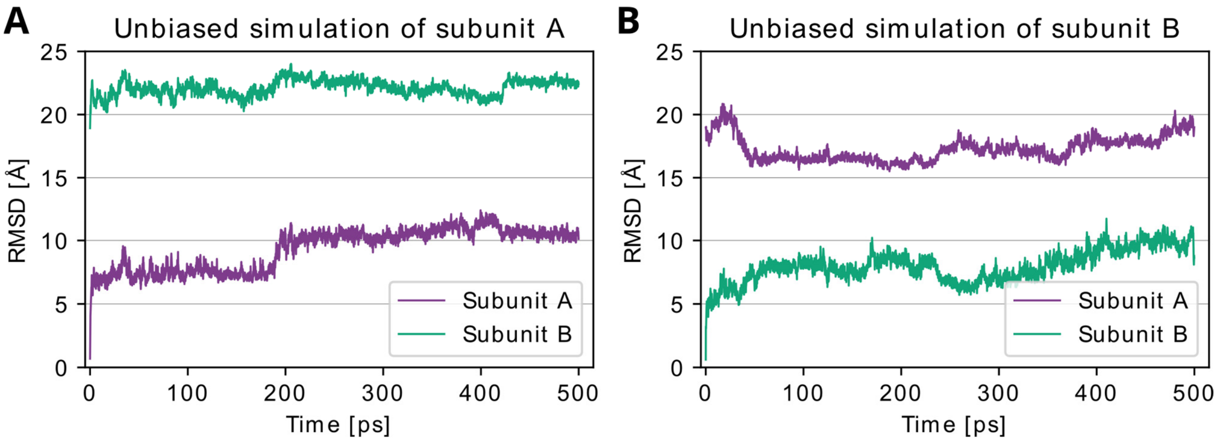

Figure S13. RMSD values of the unbiased MD simulations of monomeric MnmE in relation to subunit A (violet) and subunit B (teal). (**A**) Simulation that was started from the subunit A conformation. (**B**) Simulation that was started from the subunit B conformation.

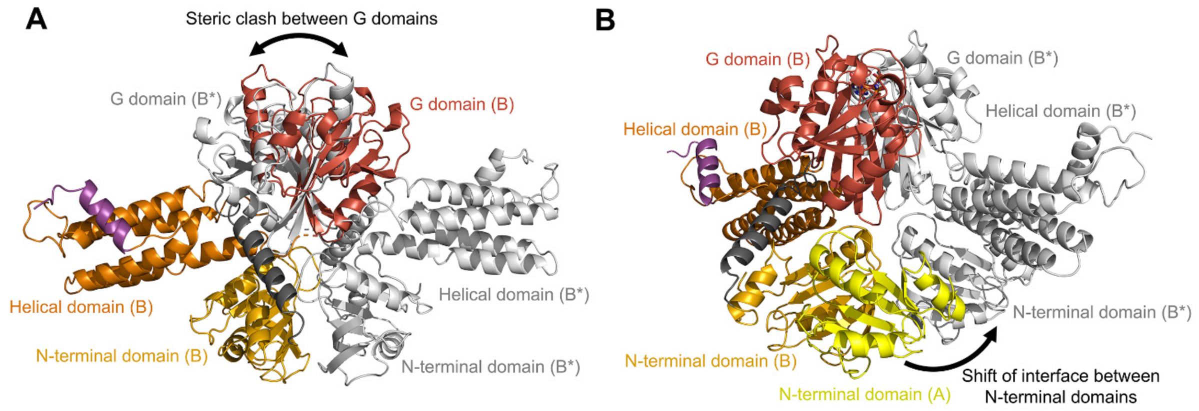

Figure S14. MnmE is unlikely to form a symmetrical dimer, in which both subunits adopt a conformation disposed to interact with MnmG ("B subunit conformation"), within the $\alpha_2\beta_2$ or $\alpha_4\beta_2$ complexes. (**A**) Creation of a symmetrical B-B* MnmE dimer, while maintaining the interface between the N-terminal domains, would result in severe clashes of the G-domains of the adjacent subunits. (**B**) Creation of a symmetrical B-B* MnmE dimer, while maintaining the interface between the G domains, would require a completely different interaction interface between the N-terminal domains of the constitutive MnmE dimer. The domains of the MnmE subunit B are colored similarly to Figure 3A. The symmetry variant of subunit B (B*) is shown in grey. In panel (B), the N-terminal domain of MnmE subunit A is shown in yellow.

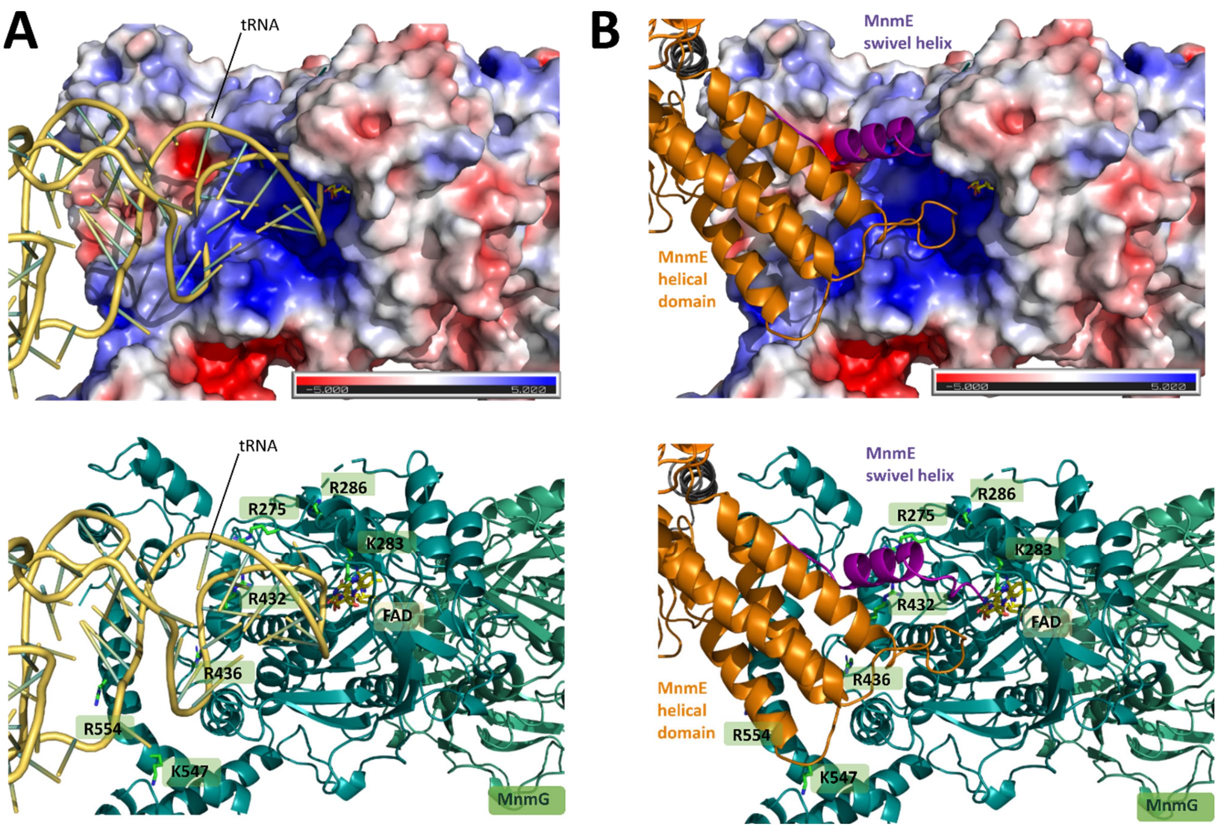

Figure S15. The MnmE and tRNA binding sites of MnmG overlap. (A) In the *top panel* MnmG from the MnmE-MnmG cryo-EM structure model ($\alpha_2\beta_2$ complex) is shown in electrostatic potential surface representation, overlaid with the tRNA molecule taken from a previously generated SAXS-based docking model of the AaMnmG-tRNA complex (yellow cartoon) [1]. The same arrangement is represented in the *bottom panel* with MnmG shown in cartoon representation (green) and key residues involved in tRNA binding indicated. **(B)** In the *top panel* the MnmE-MnmG cryo-EM structure model ($\alpha_2\beta_2$ complex) is shown, with MnmG in the same orientation and representation as in (A), while MnmE is shown in cartoon representation with its helical domain colored orange and swivel helix purple. The same arrangement is represented in the *bottom panel* with MnmG shown in cartoon representation (green) and key residues involved in tRNA binding indicated. See **Table S3 f**or a full list of residues found to be involved in tRNA binding and modification.

SUPPLEMENTARY TABLES

Table S1. Cryo-EM data collection, refinement and validation statistics.

	MnmE-MnmG $\alpha_2\beta_2$ (EMD-52197) (PDB 9HIP)	MnmE-MnmG $\alpha_4\beta_2$ (EMD-52198) (PDB 9HIQ)	MnmG focused $\alpha_4\beta_2$ (EMD-52199) (PDB 9HIR)
**Data collection**			
Microscope	CryoARM300		
Voltage (kV)	300		
Electron exposure (e–/Å^2)	62		
Energy filter slit width	20 eV		
Detector	Gatan K3		
Magnification	60,000		
Defocus range (μm)	0.5 – 3		
Pixel size (Å)	0.7596		
Initial particles (no.)	2,112,562		
Symmetry imposed	C1	C2	C1
Final particles (no.)	116,023	53,595	107,190
Map mean resolution (Å)	3.31	4.02	4.12
FSC threshold	0.143	0.143	0.143
**Refinement**			
Model composition			
Non-hydrogen atoms	15820	23248	9774
Protein residues	2043	3029	1244
Ligands	3	6	2
R.m.s. deviations			
Bond lengths (Å)	0.002	0.004	0.004
Bond angles (°)	0.416	0.532	0.673
Validation			
MolProbity score	2.53	2.33	1.9
Clash score	43.87	34.4	13.49
Poor rotamers (%)	0.55	0	0
Ramachandran plot			
Favored (%)	93.94	95.57	96.12
Allowed (%)	5.96	4.43	3.88
Disallowed (%)	0.10	0	0
B factors (Å^2)			
Protein	63.15	160.04	70.92
Ligand	70.73	165.52	65.03

Table S2. Overview of the currently described disease mutations occurring in human MTO1 and GTPBP3, together with the corresponding residues number in *E. coli* MnmG and MnmE. For the alignment of the amino acid sequences of *E. coli* MnmE and MnmG with human GTPBP3 and MTO1, respectively, the RCSB-PDB pairwise structure alignment tool was used [2]. Only point variants are included in the table, deletions or mutations causing early termination of translation are not included.

Disease mutation in MTO1 / GTPBP3	*E. coli* MnmE / MnmG residue numbering	Phenotype	Reference
**MTO1 (EcMnmG)**			
V41G	I11	COXPD10	O'Byrne et al., 2018 [3]
G59A	G29	HCM	Baruffini et al., 2013 [4]
G85R	G55	MCM	Monda et al., 2023 [5] / Kamps et al., 2018 [6]
R117H	S87	CSVD	Dunn et al., 2022 [7]
G211D	G173	COXPD10	O'Byrne et al., 2018 [3]
P241L	P203	COXPD10	O'Byrne et al., 2018 [3]
H256R	Q218	COXPD10	O'Byrne et al., 2018 [3]
T308A	E270	HCM	Baruffini et al., 2013 [4]
R313Q	R275†	MCM	Kamps et al., 2018 [6]
K321N	K283†	COXPD10	O'Byrne et al., 2018 [3]
T352M	S315	MCM	Monda et al., 2023 [5] / Luo et al., 2021 [8]
I433F*	I371	COXPD10	O'Byrne et al., 2018 [3]
T436I*	T374	COXPD10<br>HCM	O'Byrne et al., 2018 [3] / Zhou et al., 2022 [9] / Taylor et al., 2014 [10] / Baruffini et al., 2013 [4]
G450R*	G388	COXPD10	O'Byrne et al., 2018 [3]
A453T*	A391	COXPD10<br>HCM	O'Byrne et al., 2018 [3] / Baruffini et al., 2013 [4] / Ghezzi et al., 2012 [11]
R456W*	L394	COXPD10	Zhou et al., 2022 [9]
R489C*	R427†	COXPD10<br>ONCE	O'Byrne et al., 2018 [3] / Zhou et al., 2022 / Martin et al., 2017 [12]
R498C*	R436†	COXPD10	O'Byrne et al., 2018 [3]
R502C/H*	R440	COXPD10<br>HCM	O'Byrne et al., 2018 [3] / Baruffini et al., 2013 [4]
D504G	D442	COXPD10	O'Byrne et al., 2018 [3]
R529Q/W*	R447	COXPD10	O'Byrne et al., 2018 [3]
T675I*	T608	COXPD10	O'Byrne et al., 2018 [3]
**GTPBP3 (EcMnmE)**			
R3L (mitochondrial peptide)	None	Oxphos	Kohda et al., 2016 [13] / Kopajtich et al., 2014 [14]
C46G	G17	MCM	Tong et al., 2024 [15]
A61P	V32	HCM	Angelova et al., 2023 [16]
A138V	A106	COXPD23	Yan et al., 2021 [17]
E142K	E110	Oxphos	Kopajtich et al., 2014 [14] / Yan et al., 2021[17]
E159V	E127	Oxphos	Kopajtich et al., 2014 [14]
A162P	A130	Oxphos	Kopajtich et al., 2014 [14]

G182E	G150	COXPD23	Wang et al. 2022 [18] / Yan et al., 2021 [17]
A222G	I189	Oxphos	Kopajtich et al., 2014 [14]
E225K	Q192	Oxphos	Kopajtich et al., 2014 [14]
Q230P	I197	COXPD23	Wang et al. 2024 [19] / Yan et al., 2021 [17]
P257H	R224	Oxphos	Kopajtich et al., 2014 [14]
A322P	A289	Oxphos	Kopajtich et al., 2014 [14]
D337H	D304	Oxphos	Kopajtich et al., 2014 [14]
T400M	T361	HCM	Angelova et al., 2023 [16]
R430P	R389	Oxphos	Kopajtich et al., 2014 [14]
C440Y	A413	COXPD23	Wang et al. 2022 [18]
E459K	E421	Oxphos	Kopajtich et al., 2014 [14]

COXPD10: combined oxidative phosphorylation defect type 10
HCM: hypertrophic cardiomyopathy
MCM: mitochondrial cardiomyopathy
CSVD: cerebral small vessel disease
ONCE: Optic Neuropathy, Cardiomyopathy and Encephalopathy
COXPD23: combined oxidative phosphorylation defect type 23
Oxphos: Mitochondrial oxidative phosphorylation disorder

†Residues reoccurring in Table S3

*Certain references use a splice variant of MTO1 where the residue numbering of the human MTO1 is shifted -25 residues from the alignment used in this manuscript. Residue numbers in the table were adapted to the numbering used throughout this manuscript.

Table S3. Overview of MnmE and MnmG residues with previously reported roles in tRNA binding and / or the tRNA modification reaction.

Residue Number	Functionally important	Involved in tRNA binding	Reference
**MnmG**			
G13/G15	x		Shi et al, 2009 [20]
C47	x		Osawa et al, 2009 [21] / Bommisetti et al, 2023 [22]
N48	x		Osawa et al, 2009 [21]
G52/G53	x		Shi et al, 2009 [20]
K56	x		Osawa et al, 2009 [21]
G67/G68	x		Shi et al, 2009 [20]
R96	x		Osawa et al, 2009 [21]
G156/T157	x		Shi et al, 2009 [20]
T199	x		Osawa et al, 2009 [21]
T199/G200	x		Shi et al, 2009 [20]
T201	x		Osawa et al, 2009 [21]
T201/P202	x		Shi et al, 2009 [20]
R275	x (mildly affected)	x (mildly affected) *	Osawa et al, 2009 [21]
C277	x		Osawa et al, 2009 [21] / Bommisetti et al, 2023 [22]
K283	x	x *	Osawa et al, 2009 [21] / Shi et al, 2009 [20]
R286	x (mildly affected)	x (mildly affected) *	Osawa et al, 2009 [21]
Y308	x		Osawa et al, 2009 [21]
T375	x		Osawa et al, 2009 [21]
Y377	x (mildly affected)		Shi et al, 2009 [20]
R427	x		Osawa et al, 2009 [21]
R432	x	x *	Osawa et al, 2009 [21]
R436	x	x *	Osawa et al, 2009 / Shi et al, 2009 [20]
W484		x *	Osawa et al, 2009 [21]
R515	x		Osawa et al, 2009 [21]
K547		x *	Osawa et al, 2009 [21]
Y551	x		Osawa et al, 2009 [21]
R554	x (mildly affected)	x *	Osawa et al, 2009 [21]
E585	x		Meyer et al, 2009 [23]
K589	x		Meyer et al, 2009 [23]
**MnmE**			
E171	x		Meyer et al, 2009 [23]
D175	x		Meyer et al, 2009 [23]
E179	x		Meyer et al, 2009 [23]
G228	x		Yim et al, 2003 [24]
T251	x		Prado et al, 2013 [25]
R252	x		Prado et al, 2013 [25]
D253	x		Meyer et al, 2009 [23]; Prado et al, 2013 [25]
D270	x		Yim et al, 2003 [24]
L274	x		Prado et al, 2013 [25]
E282	x		Meyer et al, 2009 [23]; Prado et al, 2013 [25]
G285	x		Prado et al, 2013 [25]
R288	x		Martinez-Vicente et al, 2005 [26]

D338	**x**		Yim et al, 2003 [24]
C451	**x**		Yim et al, 2003 [24]

* Experiments performed with *A. aeolicus* MnmEG, residue numbers converted to *E. coli* numbering.

SUPPLEMENTARY REFERENCES

1. Fislage M, Brosens E, Deyaert E, Spilotros A, Pardon E, Loris R, Steyaert J, Garcia-Pino A, Versées W (2014) SAXS analysis of the tRNA-modifying enzyme complex MnmE/MnmG reveals a novel interaction mode and GTP-induced oligomerization. *Nucleic Acids Res* **42**: 5978–5992.
2. Bittrich S, Segura J, Duarte JM, Burley SK, Rose Y (2024) RCSB protein Data Bank: exploring protein 3D similarities via comprehensive structural alignments. *Bioinformatics* **40**: btae370.
3. O'Byrne JJ, Tarailo-Graovac M, Ghani A, Champion M, Deshpande C, Dursun A, Ozgul RK, Freisinger P, Garber I, Haack TB, et al. (2018) The genotypic and phenotypic spectrum of MTO1 deficiency. *Mol Genet Metab* **123**: 28–42.
4. Baruffini E, Dallabona C, Invernizzi F, Yarham JW, Melchionda L, Blakely EL, Lamantea E, Donnini C, Santra S, Vijayaraghavan S, et al. (2013) MTO1 mutations are associated with hypertrophic cardiomyopathy and lactic acidosis and cause respiratory chain deficiency in humans and yeast. *Hum Mutat* **34**: 1501–1509.
5. Sahu MR, Mondal AC (2021) Neuronal Hippo signaling: From development to diseases. *Dev Neurobiol* **81**: 92–109.
6. Kamps R, Szklarczyk R, Theunissen TE, Hellebrekers DMEI, Sallevelt SCEH, Boesten IB, De Koning B, Van Den Bosch BJ, Salomons GS, Simas-Mendes M, et al. (2018) Genetic defects in mtDNA-encoded protein translation cause pediatric, mitochondrial cardiomyopathy with early-onset brain disease. *Eur J Hum Genet* **26**: 537–551.
7. Dunn PJ, Harvey NR, Maksemous N, Smith RA, Sutherland HG, Haupt LM, Griffiths LR (2022) Investigation of Mitochondrial Related Variants in a Cerebral Small Vessel Disease Cohort. *Mol Neurobiol* **59**: 5366–5378.
8. Luo Q, Wen X, Zhou J, Chen Y, Lv Z, Shen X, Liu J (2021) A novel compound heterozygous mutation of the MTO1 gene associated with complex oxidative phosphorylation deficiency type 10. *Clin Chim Acta* **523**: 172–177.
9. Zhou C, Wang J, Zhang Q, Yang Q, Yi S, Shen Y, Luo J, Qin Z (2022) Clinical and genetic analysis of combined oxidative phosphorylation defificiency-10 caused by MTO1 mutation. *Clin Chim Acta* **526**: 74–80.
10. Taylor RW, Pyle A, Griffin H, Blakely EL, Duff J, He L, Smertenko T, Alston CL, Neeve VC, Best A, et al. (2014) Use of whole-exome sequencing to determine the genetic basis of multiple mitochondrial respiratory chain complex deficiencies. *JAMA* **312**: 68–77.
11. Ghezzi D, Baruffini E, Haack TB, Invernizzi F, Melchionda L, Dallabona C, Strom TM, Parini R, Burlina AB, Meitinger T, et al. (2012) Mutations of the mitochondrial-tRNA modifier MTO1 cause hypertrophic cardiomyopathy and lactic acidosis. *Am J Hum Genet* **90**: 1079–1087.
12. Valny M, Honsa P, Kirdajova D, Kamenik Z, Anderova M (2016) Tamoxifen in the Mouse Brain: Implications for Fate-Mapping Studies Using the Tamoxifen-Inducible Cre-loxP System. *Front Cell Neurosci* **10**: 243.
13. Kohda M, Tokuzawa Y, Kishita Y, Nyuzuki H, Moriyama Y, Mizuno Y, Hirata T, Yatsuka Y, Yamashita-Sugahara Y, Nakachi Y, et al. (2016) A Comprehensive Genomic Analysis Reveals the Genetic Landscape of Mitochondrial Respiratory Chain Complex Deficiencies. *PLoS Genet* **12**: e1005679.
14. Kopajtich R, Nicholls TJ, Rorbach J, Metodiev MD, Freisinger P, Mandel H, Vanlander A, Ghezzi D, Carrozzo R, Taylor RW, et al. (2014) Mutations in GTPBP3 cause a mitochondrial translation defect associated with hypertrophic cardiomyopathy, lactic acidosis, and encephalopathy. *Am J Hum Genet* **95**: 708–720.
15. Tong Q, Miao Y, Yin H (2024) Echocardiographic manifestations of mitochondrial disease with GTPBP3 gene mutations: A case report. *Med (United States)* **103**: E37847.

16. Angelova P, Velchev V, Stoyanov N, Atemin S, Todorov T, Tourtourikov I, Mitev V, Todorova A (2023) Novel insights on GTPBP3-associated hypertrophic cardiomyopathy. *Am J Med Genet Part A* **191**: 1804–1813.
17. Yan HM, Liu ZM, Cao B, Zhang VW, He YD, Jia ZJ, Xi H, Liu J, Fang F, Wang H (2021) Novel Mutations in the GTPBP3 Gene for Mitochondrial Disease and Characteristics of Related Phenotypic Spectrum: The First Three Cases From China. *Front Genet* **12**: 611226.
18. Wang C, Yuan C, Ji Z, Yin J, Zhang Z, Zhang H, Zheng B, Zhou W, Yang S (2022) Generation of patient-derived IPSC lines from a girl with Combined Oxidative Phosphorylation Deficiency 23 (COXPD23) caused by compound heterozygous GTPBP3 variants. *Stem Cell Res* **61**: 102775.
19. Wang Y, He J, Dong F, Shou W, Feng X, Yang Y, Li C, Wang J, Li B, Xiao S (2024) A novel mutation in GTPBP3 causes combined oxidative phosphorylation deficiency 23 by affecting pre-mRNA splicing. *Heliyon* **10**: e27199.
20. Shi R, Villarroya M, Ruiz-Partida R, Li Y, Proteau A, Prado S, Moukadiri I, Benítez-Páez A, Lomas R, Wagner J, et al. (2009) Structure-function analysis of Escherichia coli MnmG (GidA), a highly conserved tRNA-modifying enzyme. *J Bacteriol* **191**: 7614–7619.
21. Osawa T, Ito K, Inanaga H, Nureki O, Tomita K, Numata T (2009) Conserved Cysteine Residues of GidA Are Essential for Biogenesis of 5-Carboxymethylaminomethyluridine at tRNA Anticodon. *Structure* **17**: 713–724.
22. Bommisetti P, Bandarian V (2023) Insights into the Mechanism of Installation of 5-Carboxymethylaminomethyl Uridine Hypermodification by tRNA-Modifying Enzymes MnmE and MnmG. *J Am Chem Soc* **145**: 26947–26961.
23. Meyer S, Wittinghofer A, Versées W (2009) G-Domain Dimerization Orchestrates the tRNA Wobble Modification Reaction in the MnmE/GidA Complex. *J Mol Biol* **392**: 910–922.
24. Yim L, Martínez-Vicente M, Villarroya M, Aguado C, Knecht E, Armengod ME (2003) The GTpase activity and C-terminal cysteine of the Escherichia coli MnmE protein are essential for its tRNA modifying function. *J Biol Chem* **278**: 28378–28387.
25. Prado S, Villarroya M, Medina M, Armengod ME (2013) The tRNA-modifying function of MnmE is controlled by post-hydrolysis steps of its GTPase cycle. *Nucleic Acids Res* **41**: 6190–6208.
26. Martínez-Vicente M, Yim L, Villarroya M, Mellado M, Pérez-Payá E, Björk GR, Armengod ME (2005) Effects of mutagenesis in the switch I region and conserved arginines of Escherichia coli MnmE protein, a GTPase involved in tRNA modification. *J Biol Chem* **280**: 30660–30670.
