# Supplementary material for: Cryo-EM structures of the MnmE–MnmG complex reveal large conformational changes and provide new insights into the mechanism of tRNA modification
Source: Nucleic Acids Res. 2025 Aug 30;53(16):gkaf824. doi: 10.1093/nar/gkaf824 (PMC12397908; doi:10.1093/nar/gkaf824)
Supplement: gkaf824_Supplemental_Files [file gkaf824_supplemental_files.zip › Supplementary_Movie_1_legend.docx]

**Supplementary Movie 1:** Biased molecular dynamics simulation showing a possible transition pathway between the MnmE subunit A and subunit B conformations.
